# The *Plasmodium berghei* translocon of exported proteins reveals spatiotemporal dynamics of tubular extensions

**DOI:** 10.1038/srep12532

**Published:** 2015-07-29

**Authors:** Joachim M. Matz, Christian Goosmann, Volker Brinkmann, Josephine Grützke, Alyssa Ingmundson, Kai Matuschewski, Taco W. A. Kooij

**Affiliations:** 1 Parasitology Unit, Max Planck Institute for Infection Biology, Charitéplatz 1, 10117 Berlin, Germany; 2 Microscopy Core Facility, Max Planck Institute for Infection Biology, Charitéplatz 1, 10117 Berlin, Germany; 3 Institute of Biology, Humboldt University, 10117 Berlin, Germany; 4 Department of Medical Microbiology, Radboud Institute for Molecular Life Sciences, Radboud University Medical Centre, P.O. Box 9101, 6500 HB Nijmegen, The Netherlands; 5 Centre for Molecular and Biomolecular Informatics, Radboud Institute for Molecular Life Sciences, Radboud University Medical Centre, P.O. Box 9101, 6500 HB Nijmegen, The Netherlands

## Abstract

The erythrocyte is an extraordinary host cell for intracellular pathogens and requires extensive remodelling to become permissive for infection. Malaria parasites modify their host red blood cells through protein export to acquire nutrients and evade immune responses. Endogenous fluorescent tagging of three signature proteins of the *Plasmodium berghei* translocon of exported proteins (PTEX), heat shock protein 101, exported protein 2 (EXP2), and PTEX88, revealed motile, tubular extensions of the parasitophorous vacuole that protrude from the parasite far into the red blood cell. EXP2 displays a more prominent presence at the periphery of the parasite, consistent with its proposed role in pore formation. The tubular compartment is most prominent during trophozoite growth. Distinct spatiotemporal expression of individual PTEX components during sporogony and liver-stage development indicates additional functions and tight regulation of the PTEX translocon during parasite life cycle progression. Together, live cell imaging and correlative light and electron microscopy permitted previously unrecognized spatiotemporal and subcellular resolution of PTEX-containing tubules in murine malaria parasites. These findings further refine current models for *Plasmodium*-induced erythrocyte makeover.

The pathogenic features of a malaria infection are caused exclusively by repeated asexual blood-stage replication of *Plasmodium* parasites^1^. Within the erythrocyte, the parasite resides inside a membrane-bound compartment called the parasitophorous vacuole (PV), which is both protective and restrictive. Host cell remodelling is most prominent during asexual intra-erythrocytic development^2^,^3^, but occurs in all intracellular life cycle stages, including gametocytes and liver stages^4^. Since the erythrocyte is devoid of organelles, vesicular transport, and some essential nutrients, the malaria parasite needs to perform extensive remodelling to render its new home permissive for successful intracellular replication^5^.

Early morphological evidence for erythrocyte remodelling in human malarial parasites^6^,^7^,^8^ inspired extensive research to gain a better molecular and cellular understanding of the underlying mechanisms. It was not until nearly a century later that confocal microscopy allowed the visualization of a tubovesicular network forming extensive membranous structures that originate from the PV^9^,^10^. These structures have been implicated in nutrient acquisition and protein trafficking^11^,^12^, but the subsequent identification of a signature sequence in exported virulence factors, termed vacuolar transport signal (VTS)^13^ or *Plasmodium* export element (PEXEL)^14^, implied the presence of a PV membrane-resident protein translocon.

A candidate protein transport complex has been identified in *Plasmodium falciparum* and was termed the *Plasmodium* translocon of exported proteins (PTEX)^15^. Five components are thought to form a macromolecular complex. Exported protein 2 (EXP2, PBANKA_133430) is a small membrane-associated protein^16^ that likely forms the membrane-spanning pore by multimerization^17^. Heat shock protein 101 (HSP101, PBANKA_093120) is a member of the ClpA/B chaperone family and might unfold cargo proteins, a process required for *Plasmodium* protein export^18^, thereby feeding them into the central channel using the energy generated by its two AAA + ATPase domains. The biochemical functions of the three additional factors, PTEX150 (PBANKA_100850), PTEX88 (PBANKA_094130), and thioredoxin 2 (TRX2, PBANKA_135800), are less obvious.

Experimental genetics in the murine malaria model parasite *Plasmodium berghei* consistently showed that EXP2, HSP101, and PTEX150 are refractory to targeted gene deletion^19^,^20^. Using advanced knock-down technology, two studies recently reported compelling evidence for direct roles of HSP101 and PTEX150 in protein export in *P. berghei in vivo* and cultured *P. falciparum* parasites^21^,^22^. Together, all available data are consistent with a role of the PTEX complex in trafficking of virulence factors. However, the spatiotemporal development of the translocon during asexual blood infection and life cycle progression during transmission of the malaria parasite by live imaging remains to be characterized.

We previously employed live imaging of fluorescently tagged, endogenous PTEX88 to localize this component to extraparasitic protrusions^20^. This finding opened the intriguing possibility that by tracing more abundant PTEX components, parasite-induced structures can be visualized throughout blood merogony and other phases of the *Plasmodium* life cycle. In this study, we performed live imaging of endogenously tagged, functional EXP2 and HSP101, and present intriguing dynamic tubular processes initiated by a eukaryotic pathogen in a terminally differentiated host cell.

## Results

### Live imaging of the *Plasmodium berghei* PTEX component HSP101 reveals dynamic tubular extensions

**Figure 1:**
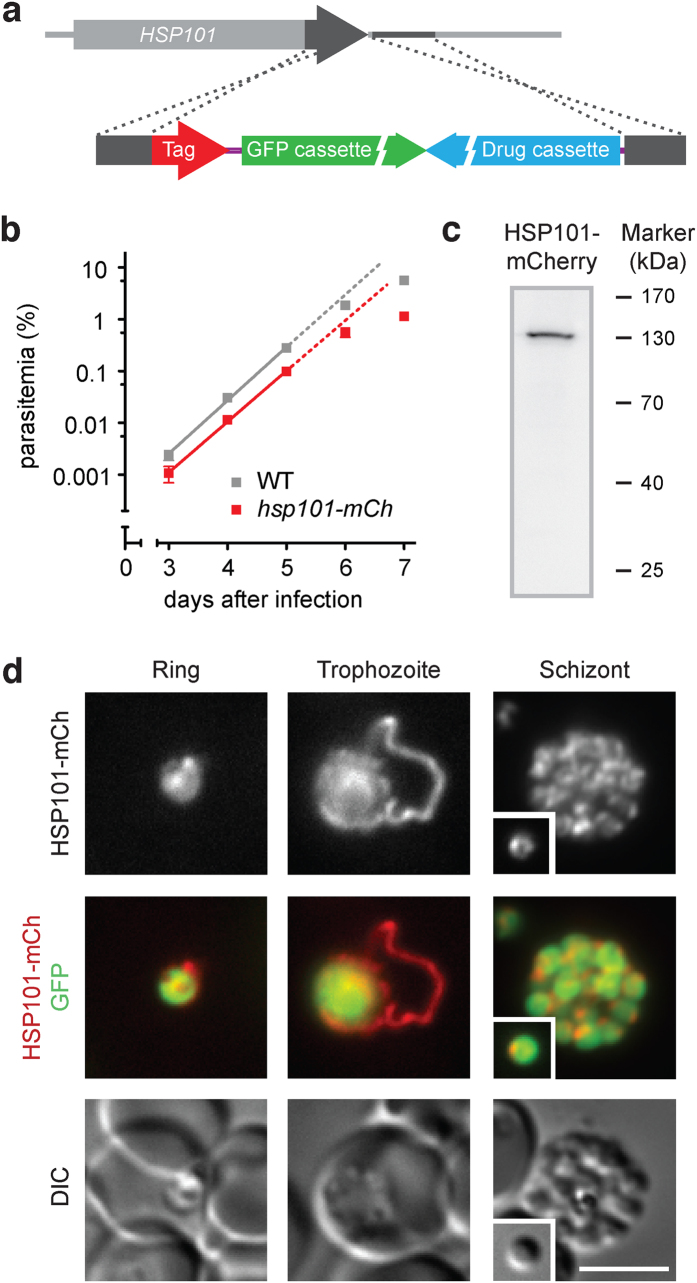
Live fluorescent imaging of *Plasmodium berghei* HSP101 during asexual blood-stage development. (**a**) Recombination strategy for the endogenous tagging of HSP101. Double crossover integration into the wild-type locus yields recombinant parasites with their endogenous locus tagged by mCherry-3xMyc. For details see Supplementary Fig. S1 and Supplementary Table S1. (**b**) Intravital competition assay of WT and *hsp101-mCherry* parasites. Parasite multiplication rates for WT and *hsp101-mCherry* parasites were 10.7 and 9.6, respectively (non-significant). (**c**) Western blot analysis of *hsp101-mCherry* parasites. The predicted size for tagged HSP101 is 133 kDa and was identified correctly using an anti-mCherry antibody. (**d**) Fluorescent micrographs of live *hsp101-mCherry*-infected erythrocytes. Shown are representative images of the fluorescent signal of HSP101-mCherry (top), a merge of HSP101-mCherry and cytoplasmic GFP (middle), and differential interference contrast images (DIC, bottom) for three asexual developmental stages. Inset, free merozoite; scale bar, 5 μm.

We initiated our analysis by generating a transgenic *P. berghei* line that contains a fluorescent mCherry-3xMyc tag fused to endogenous HSP101 (Fig. 1a and Supplementary Fig. S1). Since *HSP101* is refractory to targeted gene deletion^19^,^20^, successful selection of recombinant parasites with the desired gene replacement (Supplementary Fig. S1) and a normal parasite multiplication rate of *hsp101-mCherry* parasites during blood infection (Fig. 1b) provide direct proof for normal functioning of tagged HSP101. In addition, Western blot analysis revealed expression of the tagged protein at the expected size (Fig. 1c).

Live imaging of *hsp101-mCherry*-infected erythrocytes revealed that HSP101-mCherry switches its localization from peripheral accumulations in ring stage to one or more tubular structures during the trophozoite stage (Fig. 1d). These tubular structures emerge from the surface of the parasite and arch across the erythrocyte cytoplasm displaying vivid motility and exerting undirected folding movements (Supplemental Video S1). In mature schizonts, HSP101-mCherry localizes to peripheral foci of daughter merozoites (Fig. 1d), in good agreement with previous immunofluorescence data^15^,^17^.

**Figure 2:**
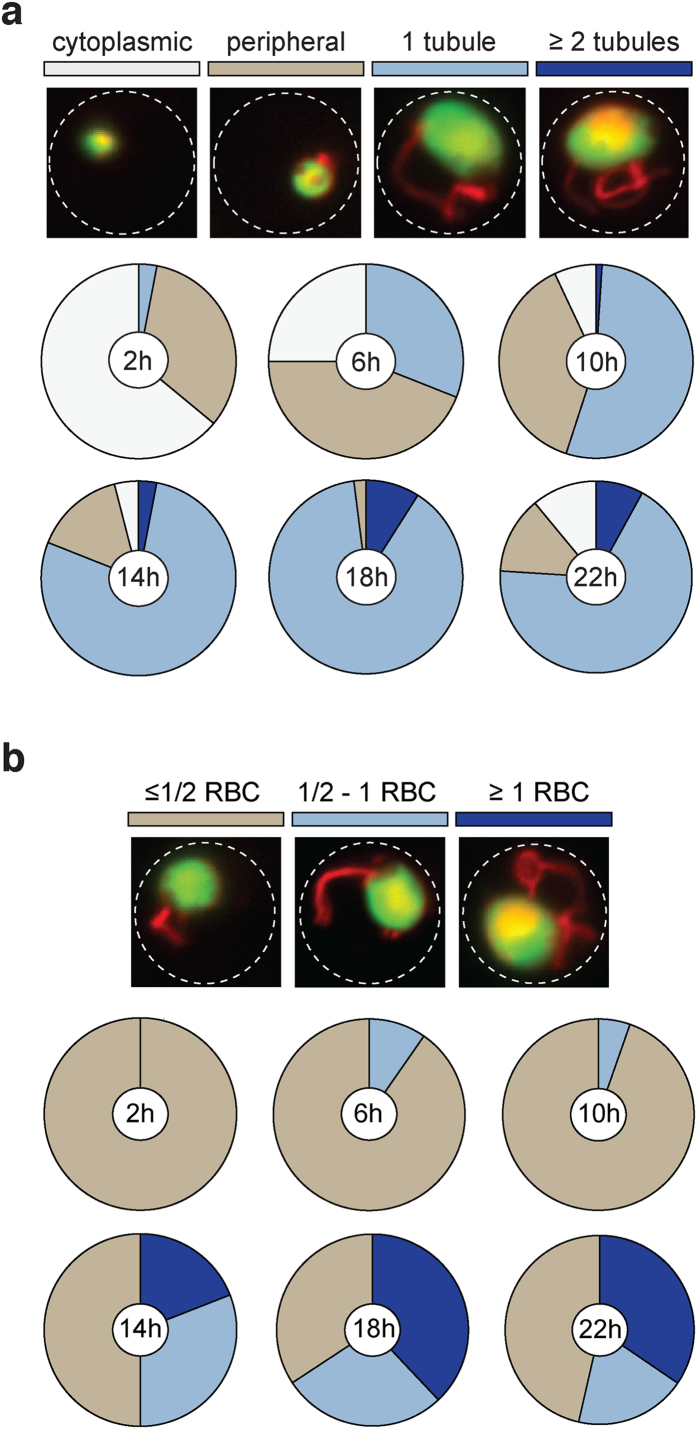
Spatiotemporal analysis of extraparasitic HSP101. (**a**) Quantification of HSP101-mCherry localization throughout a synchronized infection at 4 h intervals according to four categories indicated by representative images (top). The localization categories are: punctate cytoplasmic (white), additional periphery (light brown), one tubular extension (light blue), and two or more tubular extensions (dark blue). White outlines, erythrocyte; green, parasite cytoplasm; red, HSP101-mCherry. The proportions of extraparasitic HSP101-mCherry are indicated for six time points (*n* = 100 per time point) of the 24 h asexual blood-stage cycle. (**b**) Quantification of tubular length in relation to the red blood cell (RBC) diameter indicated by representative images (top). The length categories are: ≤0.5 RBC diameter (light brown), 0.5–1 RBC diameter (light blue), and ≥1 RBC diameter (dark blue). White outlines, erythrocyte; green, parasite cytoplasm; red, HSP101-mCherry. Length distributions of extraparasitic HSP101-mCherry are indicated for the same six time points (*n* = 100 per time point) as in (**a**).

To assess the development of the tubular structures *in vivo*, we first synchronized infections and quantified the structures by epifluorescence microscopy of tail blood samples fixed immediately following collection (Fig. 2a). Since the structures were not preserved following standard fixation protocols using methanol, acetone, or 4% paraformaldehyde, we explored a variety of different procedures and found that the structures observed during live cell imaging were preserved best following fixation with 2.5% glutaraldehyde and 4% paraformaldehyde in PBS. Several attempts to enhance the signal using anti-mCherry or anti-c-Myc antibodies were unsuccessful under these conditions, thus rendering immuno-fluorescence and -electron microscopical analyses impossible. Systematic epifluorescence analysis of the endogenously tagged HSP101 revealed that length and frequency of the tubular compartment increase during the maturation of trophozoites, peaking 18 h after invasion, and eventually decrease when reaching the schizont stage (Fig. 2).

### HSP101 is trafficked by the parasite secretory pathway

We postulated that the dynamic structures stained by HSP101-mCherry are motile evaginations of the PV, while the intraparasitic proportion of tagged protein (Fig. 1d) localizes to the endoplasmic reticulum (ER). We confirmed these findings using double mutant *P. berghei* strains that were generated by two rounds of advanced genetic manipulation. We first generated parasites with fluorescently labelled endogenous HSP101 that lack both GFP and drug-selectable cassette (Supplementary Fig. S1). In this *hsp101-mCherry* line, we introduced a transgenic GFP marker fused to the signal peptide and ER-retention sequences of *Pb*HSP70-2/BiP (PBANKA_081890), which labels the parasite’s ER (GFP^ER^; Supplementary Fig. S2). Live imaging of the resulting *hsp101-mCherry*/*GFP*^ER^ line revealed co-localization of GFP^ER^ with internal HSP101-mCherry signal (Supplemental Fig. S3a).

To further test whether localization of HSP101-mCherry to the tubular structures depends on the parasite’s secretory pathway, we inhibited transport from the ER onward with brefeldin A (BFA), an ARF guanine nucleotide exchange inhibitor (Supplemental Fig. S3b). As expected, inhibition of infected erythrocytes with BFA resulted in accumulation of the fluorescent signal inside the parasite, presumably the ER.

### HSP101-positive tubules originate from the parasitophorous vacuole

**Figure 3:**
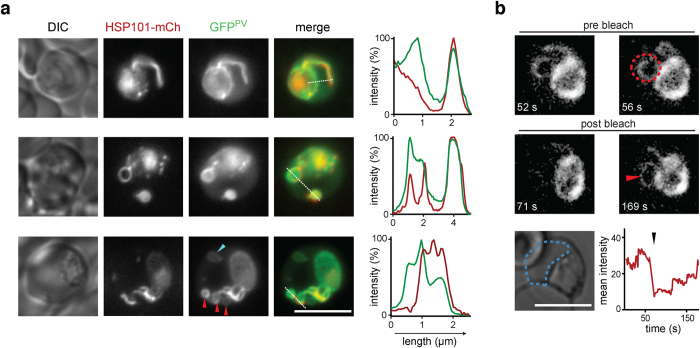
HSP101 delineates a tubular subcompartment of the parasitophorous vacuole. (**a**) Live co-localization of HSP101-mCherry (centre left) with a marker protein of the parasitophorous vacuole (GFP^PV^, centre). The line in the merge (centre right) indicates profiling of the fluorescent signal (right). Shown are three representative trophozoites demonstrating vacuolar tubules, loops, and vesicles. The blue arrowhead denotes a detached, vacuole-derived, and HSP101-mCherry negative lumen. The red arrowheads denote budding structures at the site of a vacuolar tubule. Note that HSP101-mCherry is excluded from these compartments. (**b**) FRAP analysis reveals free diffusion from the parasitophorous vacuole to the tubular extensions. Erythrocytes infected with *mCherry*^PV^ parasites were analysed by confocal microscopy before (pre bleach) and after (post bleach) photo bleaching (red area, bleach location). Shown is a representative trophozoite and the respective temporal fluorescence analysis in the erythrocyte cytoplasm (blue dotted line); black arrowhead indicates time of the bleaching pulse. Scale bar, 5 μm.

Since HSP101 harbours a signal peptide and is trafficked by the parasite’s secretory pathway, we postulated that the dynamic structures are motile evaginations of the PV. In order to test this hypothesis, we introduced a different transgenic GFP marker fused only to the HSP70-2/BiP signal peptide sequence, which labels the parasite’s PV (GFP^PV^), into *hsp101-mCherry* parasites (Supplementary Fig. S2). Live imaging revealed near-perfect co-localization of GFP^PV^ with tubular HSP101-mCherry (Fig. 3a). As anticipated, the PV marker is not restricted to the tubular extension but also shows a typical peripheral distribution around the parasite, from which HSP101-mCherry is excluded in mature blood stages. In addition, we observed low-level signal from the erythrocyte cytoplasm indicating some leaking of this abundant marker protein.

Though the typical appearance of double labelled mature trophozoites constituted a single GFP^PV^- and HSP101-mCherry-positive tubule, we observed a diversity of less regular patterns (Fig. 3a). HSP101-positive protrusions occasionally formed large loops, which in some cases were filled with GFP^PV^ signal. These dually labelled vesicles were also observed detached, suggesting that the tubular extensions might act as sites of membrane budding. Moreover, we captured loops and vesicles that were stained with the PV marker, but which were negative for HSP101-mCherry, suggestive of potential sub-compartmentalization of the PV-derived extensions.

### Free protein exchange between the PV and tubules

To better understand the connectivity between the PV and the tubular extensions, we examined the ability of a *P. berghei* PV-marker to diffuse between these compartments by fluorescence recovery after photobleaching (FRAP). For such an analysis, the *hsp101-mCherry* parasite line was not suitable due to its exclusive localization to the tubular extensions. As an alternative, we employed a parasite line, which, like *GFP*^PV^, localizes to the extraparasitic tubular extensions and peripheral to the blood-stage parasites. The reporter consists of an amino-terminal fragment of the exported protein IBIS1 (PBANKA_136550)^23^, which is insufficient for export into the host cell, fused to mCherry (Supplementary Fig. S2). The *mCherry*^PV^ line was preferred over the *GFP*^PV^ line due to its stronger and more stable fluorescence signal, rendering it particularly suited for confocal imaging. When the mCherry^PV^ signal was bleached in the tubules, we consistently observed signal recovery (Fig. 3b, Supplementary Fig. S4, and Supplementary Videos S2 and S3). We conclude that (i) the lumen of the tubular extensions is contiguous with the PV and (ii) the tubular structures contain a specific protein composition, distinct from the residual PV.

### HSP101-positive tubules are membrane-bound and present in wild-type parasites

**Figure 4:**
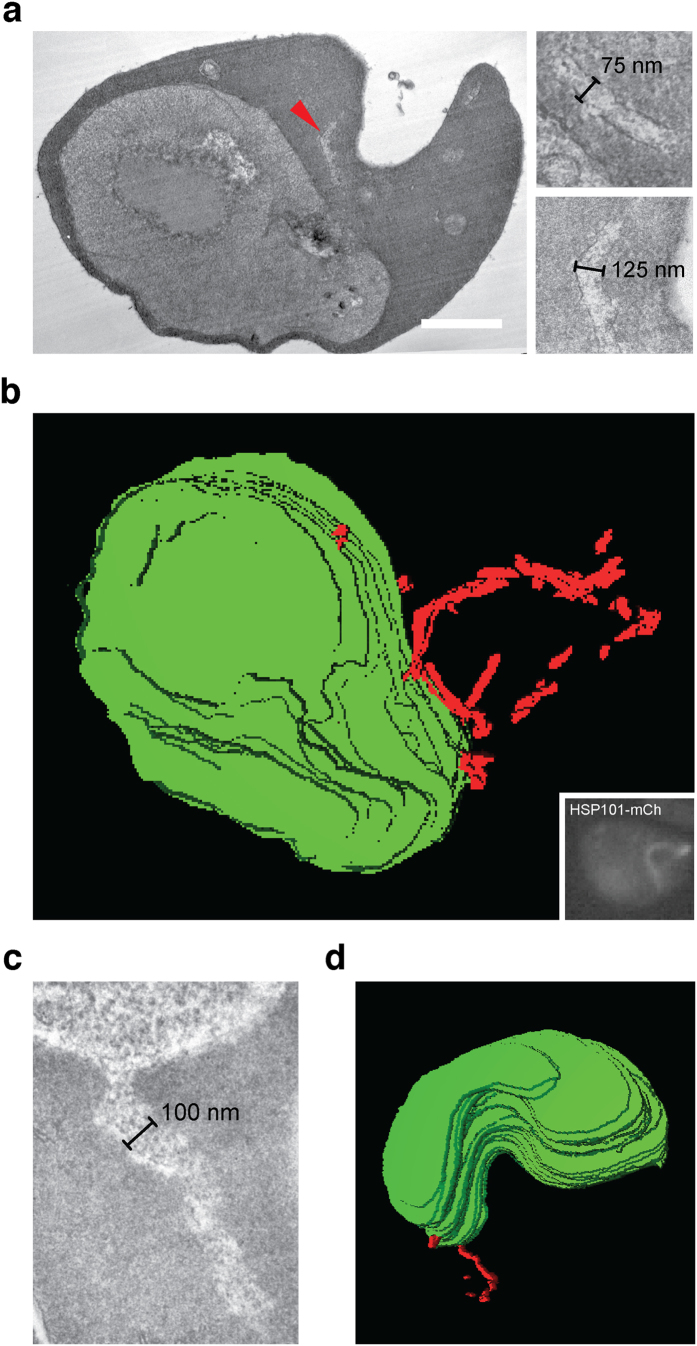
Ultrastructure of the vacuolar tubules. (**a**) Representative transmission electron micrograph (TEM) of an *hsp101-mCherry*-infected erythrocyte, obtained by correlative light and electron microscopy (left). The red arrowhead denotes a tubular extension. Two representative high magnification images of the compartment are shown (right). Scale bar, 1 μm. (**b**) Tubules were visualized by fluorescence microscopy (inset) and correlated with multiple transmission electron microscopic (TEM) sections of the same cell. The 3D-reconstruction was generated by parasite membrane alignment of 29 consecutive TEM sections. Green, parasite surface; red, tubule. (**c**) TEM section of a WT-infected erythrocyte. Shown is a representative high magnification image of a vacuolar tubule. (**d**) 3D-reconstruction generated by parasite membrane alignment of 19 TEM sections of a WT-infected erythrocyte. Green, parasite surface; red, tubule.

To characterize the tubular ultrastructure in *P. berghei*-infected erythrocytes, we employed correlative light and electron microscopy (CLEM) using *hsp101-mCherry*-infected erythrocytes (Fig. 4a,b). We were able to correlate the fluorescent HSP101-mCherry signal with continuous extended membrane evaginations (Fig. 4a), which are distinct from intra-erythrocytic *P. berghei*-induced structures (IBIS), previously identified by correlative light and electron microscopy of *IBIS1-mCherry*-infected erythrocytes^23^. While the latter could be assigned to punctate structures that correlate with short membranous tubules found scattered across the erythrocyte cytoplasm, the tubules appeared much more elongated and wider in diameter.

3D reconstruction of the micrographs revealed a tubular compartment with a variable diameter of ∼100 nm (75–125 nm; Fig. 4a,b and Supplementary Video S4). The protrusions appear to be confined by a singular membrane. Close examination of the tubular lumen revealed a uniform transparent appearance, indicative of soluble rather than filamentous content.

To further exclude the contribution of a cytoskeleton to the motility of the tubules, we tested a range of inhibitors of tubulin and actin filament polymerization, *i.e.* nocodazole, cytochalasin D, and jasplakinolide, as well as motility inhibitors, *i.e.* blebbistatin, erythro-9-(2-hydroxy-3-nonyl)adenine (EHNA), and vanadate. None of the tested inhibitors affected motility or appearance of the HSP101-positive extensions (Supplementary Table S2).

Previous work showed that the dye BODIPY TR ceramide delineates a tubovesicular network (TVN) in *P. falciparum*^9^. In order to test whether this dye displays a similar signal in *P. berghei*-infected erythrocytes, we added BODIPY TR ceramide to erythrocytes infected with *GFP*^PV^ parasites (Supplementary Fig. S5). We detected, albeit irregular and weak, signals, which occasionally coincided with GFP^PV^-positive structures. This co-localization was particularly prominent in loop structures (Supplementary Fig. S5). The TVN-specific inhibitor DL-*threo*-1-phenyl-2-palmitoylamino-3-morpholino-1-propanol (PPMP)^24^ did neither affect appearance nor motility of the protrusions in *P. berghei* parasites (Supplementary Table S2). Despite apparent differences, the overall striking similarities suggest that the HSP101-positive structures observed *ex vivo* in *P. berghei*-infected erythrocytes might share aspects of the TVN described in cultured *P. falciparum*-infected erythrocytes^9^,^10^,^11^,^12^.

In order to confirm the presence of a membrane-bound tubular compartment in wild-type (WT)-infected erythrocytes, we synchronized a *P. berghei* culture and scanned trophozoite-infected erythrocytes by transmission electron microscopy (Fig. 4c,d, Supplementary Fig. S6, and Supplemental Video S5). The presence of translucent membranous tubules extending from the PV further corroborated the physiological relevance of the structures detected in the *hsp101-mCherry* parasites. 3D-reconstruction of consecutive thin sections demonstrated a close association of the tubules with the PV (Fig. 4d), lending additional ultrastructural support for this tubular compartment and its connectivity to the PV in parasite-infected erythrocytes.

### Vacuolar tubules harbour at least three PTEX components

**Figure 5:**
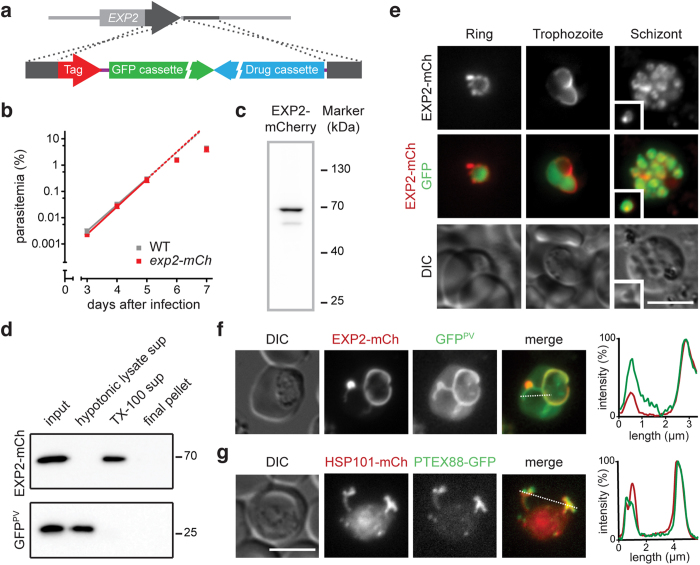
Live fluorescent imaging of the PTEX components EXP2 and PTEX88. (**a**) Recombination strategy for the endogenous tagging of EXP2. Double crossover integration into the wild-type locus yields recombinant parasites with their endogenous locus tagged by mCherry-3xMyc. (**b**) Intravital competition assay of WT and *exp2-mCherry* parasites. Parasite multiplication rates for WT and *exp2-mCherry* parasites were 10.0 and 11.2, respectively (non-significant). (**c**) Western blot analysis of *exp2-mCherry* parasites. The predicted size for tagged EXP2 is 62 kDa and was identified correctly using an anti-mCherry antibody. (**d**) Purified *exp2-mCherry* × *GFP*^PV^-infected erythrocytes were lysed with hypotonic buffer (input) and spun at 100 000 × *g*. The supernatant (hypotonic lysate sup) along with proteins released from the pellet after Triton X-100 treatment (TX-100 sup) and the remaining insoluble pellet were analyzed by SDS-PAGE and Western blotting using anti-mCherry (EXP2-mCh) and anti-GFP (GFP^PV^) antibodies. (**e**) Micrographs of live *exp2-mCherry*-infected erythrocytes. Shown are representative images for three asexual developmental stages including the fluorescent EXP2-mCherry signal (top), a merge of EXP2-mCherry and cytoplasmic GFP (middle), and differential interference contrast images (DIC, bottom). Inset, free merozoite. (**f**) Co-localization of EXP2-mCherry (centre left) with the parasitophorous vacuole (GFP^PV^, centre). The line in the merge (centre right) indicates profiling of the fluorescent signal (right). (**g**) Co-localization of HSP101-mCherry (centre left) with PTEX88-GFP (centre). The line in the merge (centre right) indicates profiling of the fluorescent signal (right). Scale bars, 5 μm.

To test whether tubular localization is a unifying feature of all *P. berghei* PTEX core components and PTEX88, we employed a strategy equivalent to the one used for HSP101. We generated recombinant parasites expressing a fluorescently labelled endogenous EXP2 protein (Fig. 5a and S1b). A normal parasite multiplication rate of *exp2-mCherry* parasites (Fig. 5b) together with the reported refractoriness of *EXP2* to targeted gene deletion^19^,^20^ and detection of a tagged protein of the expected size by Western blot analysis (Fig. 5c) indicate normal functions of this fusion protein. We also confirmed that EXP2-mCherry is only solubilized after treatment of membranes with Triton X-100, indicating that the large tag does not interfere with EXP2 insertion into membranes (Fig. 5d). Repeated attempts to endogenously tag PTEX150 were unsuccessful, indicating that the mCherry-3xMyc tag interferes with protein function (Supplementary Fig. S1b).

We next performed live imaging of *exp2-mCherry*-infected erythrocytes and compared the signal to GFP markers of the parasite cytoplasm (Fig. 5e) and the PV (Fig. 5f). In good agreement with previous findings^15^,^16^,^17^,^19^, we detected a circumferential staining pattern delineating the developing parasite during early blood-stage development (Fig. 5e). In maturing stages, the signal is concentrated to one or two particular zones, which often appear to form blebs extending away from the parasite (Fig. 5e and Supplementary Fig. S7), but always matches the pattern of the GFP^PV^ marker indicating a distribution throughout the entire PV including the vacuolar tubules where HSP101 resides (Fig. 5f). We note that both signals also label vesicles, frequently observed in the erythrocyte cytoplasm during the trophozoite stage (Supplementary Fig. S7 and Supplementary Video S6).

Confirmation that the PTEX88-positive extensions, which we reported earlier^20^, are indeed the vacuolar tubules harbouring HSP101 and EXP2 was obtained through a genetic cross of *hsp101-mCherry* and *ptex88-GFP*, a parasite line expressing PTEX88 endogenously tagged with GFP (Supplementary Fig. S1). The double fluorescent parasites displayed the exact same extraparasitic protein distribution (Fig. 5g). The exclusion from the remainder of the PV, with the exception of a few smaller foci, further strengthens the notion that the tubules are distinct from the PV. Therefore, PTEX88 forms a second signature protein of this compartment, while live imaging of EXP2-mCherry reveals two distinct localizations of this putative PTEX pore protein.

### Spatiotemporal dynamics of PTEX components during *Plasmodium berghei* life cycle progression

**Figure 6:**
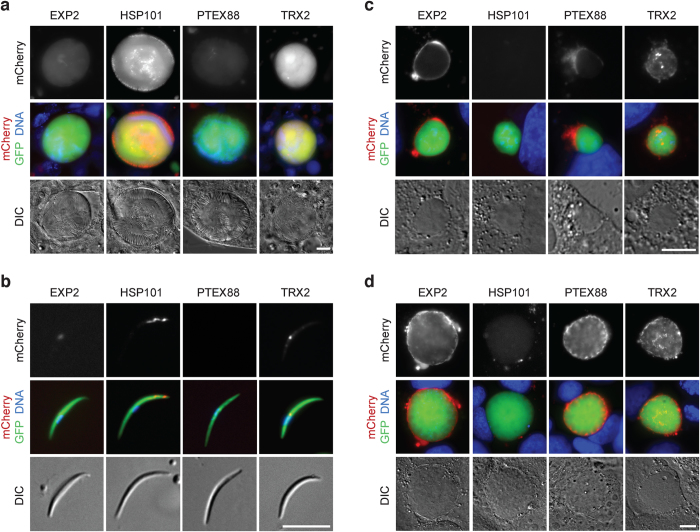
Live imaging of four PTEX components during *Plasmodium berghei* life cycle progression. Micrographs of live midgut-associated oocysts (**a**), salivary gland sporozoites (**b**), and liver stages 24 h (**c**) and 48 h after infection (**d**). Shown are representative images including the fluorescent signal of the tagged protein (top), a merge of tagged protein, cytoplasmic GFP, and Hoechst 33342 DNA dye (middle) and differential interference contrast images (DIC, bottom). Scale bars, 10 μm.

Transcription profiling of the genes believed to encode the *P. berghei* PTEX components has demonstrated that these are active almost throughout the entire life cycle^19^. Encouraged by the dynamic spatiotemporal expression and localization in live blood stage parasites, we performed a systematic analysis of the timing and localization of the four endogenously tagged proteins, HSP101, EXP2, PTEX88, and TRX2^20^ (Fig. 6).

When we examined midguts from infected *Anopheles stephensi* mosquitoes, we noted abundant expression and uniform distribution of TRX2 (Fig. 6a). EXP2 also displayed a uniform, though barely detectable red fluorescent signal, while *ptex88-mCherry* oocysts never reached levels above background seen in WT parasites (Fig. 6a). HSP101 was also readily detectable and displayed a distinct circumferential pattern in addition to uniform cytoplasmic distribution inside developing oocysts (Fig. 6a).

In mature, salivary gland sporozoites, the distinct temporal expression essentially remained, *i.e.* PTEX88 was not detectable and EXP2 showed an extremely faint, diffuse accumulation barely above background, whereas TRX2 and HSP101 signals were clearly present in individual sporozoites (Fig. 6b). Strikingly, HSP101 localized to the apical tip of sporozoites, reminiscent of the peripheral localization in free merozoites (Fig. 1d), while TRX2 localized to punctate structures inside sporozoites, as reported previously for blood-stage parasites^20^,^25^.

PTEX expression displayed a rather different pattern during liver-stage development. EXP2 and PTEX88 were continuously expressed during liver-stage maturation and localized to the periphery of the developing parasite, most likely the PV (Fig. 6c,d). TRX2 continued to be expressed in this phase of the life cycle and localized initially to multiple, intraparasitic foci, but in more mature stages also to the parasite-host interface. In marked contrast and despite its presence during development in the definitive mosquito host, HSP101 expression was completely switched off during the first two days of intrahepatic growth (Fig. 6c,d). As expected, all four PTEX components were expressed in merozoites derived from *in vitro* liver-stage cultures in preparation of a new blood infection (Supplementary Fig. S8).

Together, the distinct patterns of all four PTEX components indicate that the constellation of the translocon may vary considerably during *Plasmodium* life cycle progression. Furthermore, the different components may also fulfil additional functions unrelated to the multimeric protein complex described for asexual intra-erythrocytic propagation, *e.g.* during mosquito-stage development.

## Discussion

*Plasmodium* parasites have the remarkable ability to remodel their host cell by membrane and protein trafficking. Most of our understanding of parasite-induced erythrocyte manipulation has come from studies of the human malaria parasite *P. falciparum*^2^,^26^,^27^,^28^. Detailed electron microscopic analyses have revealed a close functional and physical association of parasite derived membranous structures, such as the Maurer’s clefts, and the cytoadhesion complex in *P. falciparum*^29^,^30^. Despite our growing insights, it remains unclear how protein export mechanisms and parasite-induced membrane-structures in the erythrocyte cytoplasm relate.

In this work, we demonstrate that components of the putative protein export translocon localize to a specific, perhaps even specialized, tubular compartment of the PV. We identified HSP101 and PTEX88, two components of the proposed *Plasmodium* translocon, as signature proteins that localize to this tubular lumen of the PV. Thus far, localization data of the PTEX components have been consistently obtained using immunofluorescence in fixed ring-stage parasites and mature schizonts or merozoites^15^,^17^,^19^,^31^. Our data are consistent with the reported apical localization in merozoites and the specific peripheral foci in ring stages. With the exception of our own live imaging of PTEX88-mCherry^20^, which revealed a similar though much weaker staining pattern as described here for HSP101-mCherry (Fig. 1), none of the previous studies reported tubular extensions. There are two reasons that may explain why the structures have remained elusive. Firstly, the tubules are fixation-sensitive and collapse unless high glutaraldehyde concentrations are applied, something that might even contribute to the previously observed “beads-on-a-string” staining pattern. Secondly, none of the published data show parasites at the second half of their intra-erythrocytic development when the tubular structures are largest and most prevalent.

Whereas the function of HSP101 in protein export has been demonstrated convincingly in both human and rodent malaria parasites^21^,^22^, PTEX88 does not appear to play a direct role in protein translocation despite being pivotal to parasite virulence^32^. However, the striking co-localization of HSP101 and PTEX88 strengthens the hypothesis that both components fulfil functions as part of the protein export complex. One open question that remains is how this co-localization is achieved at these very specific loci, particularly considering the apparent absence of filamentous structures.

EXP2, which has been hypothesized to build the membrane-spanning pore of the translocon, was also found in the PV-tubules, though not exclusively. In addition, we observed EXP2 along the parasite periphery not limited to a few specific foci, arguing for multiple functions. This pattern resembles immuno-electron microscopic observations of exported protein 1 (EXP1) that localized to the PV and extraparasitic, tubular loops in *P. falciparum*-infected erythrocytes^11^. Our observation that EXP2 also localizes to vesicles in the erythrocyte cytoplasm is supported by a concurrent publication, reporting similar vesicular EXP2-positive structures, primarily in reticulocytes infected with human or rodent malaria species^33^. Collectively, these data support the notion that a tubular network originates from the PV of the developing intra-erythrocytic parasite. The multiple localizations and differential transcription profile, render it conceivable that an EXP2-formed channel module may fulfil several transport functions, *e.g.* in protein export, waste disposal, as well as nutrient acquisition, depending on its location, protein interaction partners, or post-translational modifications.

Together with the recent identification in the rodent malaria model parasite *P. berghei* of small cleft-like structures, to which exported proteins are specifically trafficked^23^,^34^, the present characterization of dynamic PV membrane tubules highlights the universal capacity of malaria parasites to extensively remodel host erythrocytes. The presence of an extensive membranous network originating from the parasitophorous vacuole that is implicated in protein trafficking compares in many respects with the description of the *P. falciparum* TVN^9^,^10^,^11^,^12^. Indeed, the first observation of a *P. berghei* TVN was made following the expression of GFP fused to a *P. falciparum* signal peptide sequence, which led the authors speculate that these tubular structures may facilitate protein export^35^. In the context of a high-resolution localization study of *P. falciparum* PTEX components, whorl-like structures were also described that were devoid of any such components^31^. However, these structures appear within minutes of invasion and disappear soon after, whereas we observe tubular motile PV extensions predominantly in maturing trophozoites. In *P. falciparum*, the TVN has been shown to release double membrane vesicles^12^. We observed HSP101-delineated tubular loops, where the enclosed space was marked by GFP^PV^, which is consistent with the genesis of double membrane compartments. Furthermore, the TVN was described as a site in which parasite-derived proteins can be specifically enriched^9^, as demonstrated for HSP101 and PTEX88 in the present study.

Several of our observations set the described vacuolar tubules apart from the original description of the *P. falciparum* TVN: (i) the HSP101-, EXP2-, PTEX88-, and GFP^PV^-positive tubular compartment is highly dynamic, whereas the TVN was described as a rather static membrane network^10^; (ii) fixation with formaldehyde preserved membrane morphology of the TVN in *P. falciparum*-infected erythrocytes^9^, while the tubular compartment of *P. berghei* was only conserved when employing fixation with 2.5% glutaraldehyde; (iii) the sphingomyelin synthase inhibitor PPMP blocks TVN assembly in *P. falciparum*^11^,^24^, but not the development of the *P. berghei* PV-tubules, and (iv) the lipid marker BODIPY TR ceramide, which visualizes TVN membranes in *P. falciparum*, did not consistently stain the PV-tubules, despite clear visibility of the derived vesicular structures. Though many of these differences may be attributed to species-specific characteristics, additional work is necessary to confidently label the tubular protrusions as TVN. The absence of a population of HSP101-negative tubular structures as detected by CLEM supports the notion that a detailed characterization of the TVN development in *P. falciparum*-infected erythrocytes, as described herein for the murine parasite, will further highlight the parallels between mechanisms of host-cell remodeling of both species. We favour the hypothesis that the tubules along with a limited number of peripheral foci define (sub)compartments of the interconnected PV/TVN space that might have evolved to specifically serve protein export to remodel the erythrocyte, while the EXP2-positive sites devoid of HSP101 or PTEX88 specialized in other functions, *e.g.* nutrient acquisition.

In a previous study, transcripts of all PTEX translocon components were detected by non-quantitative RT-PCR throughout the entire life cycle of *P. berghei*^19^. Our live imaging analysis, however, demonstrated that several of the PTEX components were not or barely detectable at the protein level during several phases of the life cycle. Most striking is the inverse correlation of the expression levels of the two components co-localizing perfectly in blood-stage parasites, HSP101 and PTEX88. The apparent absence of HSP101 during liver-stage growth contrasts with abundant expression during parasite propagation in the *Anopheles* vector, where PTEX88 expression is not evident. EXP2, like PTEX88, is expressed in the PV of the developing liver-stage parasites, while signals in mosquito stages are only marginally above background levels. The observed protein expression levels in liver-stage parasites largely reflect the transcription levels^19^. In developing oocysts, *PTEX88* and *HSP101* transcription levels are equivalent and those of *EXP2* are even much higher, contrasting with our protein expression data. A simple explanation for the discrepancies could be the detection of leaky transcription by the sensitive PCR-based method. It is also important to note that the transcription data are not quantitative, which is reflected by different transcription levels of the *P. yoelii* orthologues in these stages^36^,^37^. Together, these data could also be indicative of post-transcriptional silencing of *PTEX* gene expression, with striking distinct patterns. Systematic studies of candidate mechanisms, such as translational repression during host switch^38^,^39^, will be important to assign functions to PTEX components throughout the *Plasmodium* life cycle. While interpretations remain speculative without functional evidence using stage-specific knock-downs, the tight and exclusive regulation of distinct PTEX components already justify the notion that these proteins fulfil additional and distinct functions in other parasite life cycle stages. Based on our data, we postulate that the apparent absence of HSP101 protein during liver-stage development offers a plausible molecular explanation for the observed retention of PEXEL/VTS proteins inside the PV during intrahepatic parasite propagation^40^,^41^.

In conclusion, this study establishes that PTEX components, which function in a macromolecular complex and primarily in protein translocation across the PV membrane, are signature proteins of PV tubules that might reflect an evolutionary conserved protein trafficking tubular system.

## Methods

### Ethics statement

This study was carried out in strict accordance with the German ‘Tierschutzgesetz in der Fassung vom 22. Juli 2009’ and the Directive 2010/63/EU of the European Parliament and Council ‘On the protection of animals used for scientific purposes’. The protocol was approved by the ethics committee of the Berlin state authority (Landesamt für Gesundheit und Soziales Berlin, permit number G0469/09). Female NMRI and C57BL/6 mice were purchased from Charles River Laboratories (Sulzfeld, Germany). NMRI mice were used for blood-stage growth assays and parasite cultivation. Sporozoite transmission was performed using C57BL/6 mice.

### Generation and isolation of recombinant *Plasmodium berghei* parasite lines

Recombinant parasite lines were generated and isolated as described^42^,^43^,^44^. Transfection plasmids designed for endogenous tagging were based on the pBAT vector^45^ and constructed following a similar strategy as described previously^20^ (Supplementary Fig. S1 and Supplementary Table S1). In a first cloning step, the 3' flanking regions of *EXP2* (606 bp) and *HSP101* (848 bp) were amplified from genomic DNA and inserted into the pBAT vector, using the XhoI and KpnI restriction sites. The resultant intermediate constructs (pEXP2-IM and pHSP101-IM) were digested with SacII and HpaI prior to insertion of the carboxy-terminal coding sequences of *EXP2* (703 bp) and *HSP101* (638 bp). In the final pEXP2-mCh and pHSP101-mCh plasmids, the carboxy-terminal sequences of the genes were thus fused in frame to an mCherry-3xMyc-tag, allowing for live protein localization in the resulting *exp2-mcherry* (*exp2-mCh*^GFP,res^) and *hsp101-mCherry* (*hsp101-mCh*^GFP,res^) parasite lines (Supplementary Fig. S1). For co-localization purposes with GFP-coupled proteins, the high-fluorescent GFP expression cassette was removed by PvuII/EcoRV digestion and plasmid re-ligation. A transfection plasmid for the generation of a parasite line expressing the endogenous *PTEX88* fused in-frame to *GFP* was generated by digesting the pPTEX88-tag plasmid^20^ with SwaI and AgeI, followed by Klenow fill-in and plasmid re-ligation.

For the generation of two novel reference strains with GFP marker proteins staining either the parasitophorous vacuole (*GFP*^PV^) or the endoplasmic reticulum (*GFP*^ER^), the GFP coding sequence of the pBAT-SIL6 plasmid was equipped with the BiP signal peptide at the amino-terminal, either alone (*GFP*^PV^, 818 bp) or in combination with a carboxy-terminal *BiP* ER retention signal (*GFP*^ER^, 830 bp). Inserted coding sequences were confirmed by commercial Sanger sequencing. All pBAT-based plasmids were linearized with ApaLI and AhdI prior to transfection and integration into the genome of *P. berghei* ANKA parasites through stable double crossover homologous recombination.

For the mCherry^PV^ plasmid, the first 484 bp of the coding sequence of *IBIS1* and 1,282 bp of the 5′ flanking region were cloned into the b3D+mCherry vector^46^, using the SacII and SpeI restriction sites. Following linearization, the mCherry^PV^ transfection vector was integrated into the genome of *P. berghei* GFPcon^42^ through single crossover homologous recombination. The lack of a spacer between the IBIS1 PEXEL/VTS motif and mCherry prevents export of the fusion protein. Successful integration of all transfection vectors into the endogenous *EXP2*, *HSP101*, *PTEX88*, and *IBIS1* loci or into the silent intergenic locus on *P. berghei* chromosome 6 (SIL6) was confirmed by diagnostic PCR (Supplementary Fig. S1 and S2 and Supplementary Table S1).

### Strategies for the generation of parasite double mutants

We followed two different strategies to generate double mutant parasite lines. (1) The isogenic pyrimethamine-insensitive but *GFP*-negative *hsp101-mCherry* (*hsp101-mCh*^res^) line was subjected to negative selection with 5-fluorocytosine, yielding *hsp101-mCh*^sens^ parasites that are accessible for a subsequent round of genetic manipulation due to the loss of their drug-selectable resistance cassette. The clonal *hsp101-mCh*^sens^ line was transfected with the pGFP^PV^ and pGFP^ER^ plasmids and images were recorded directly from the parental populations. (2) In a complementary approach, we infected NMRI mice with two parasite lines of different genetic backgrounds and fed these mice to *Anopheles stephensi* mosquitoes. Sporozoite transmission was achieved by bite back feeding of C57BL/6 mice. Cross-fertilization and chromosomal recombination during mosquito stage development yielded a mixed population of single and double mutant blood-stage parasites. This method was employed to generate genetic crosses of *exp2-mCherry * × *GFP*^PV^ and *hsp101-mCherry* × *ptex88-GFP*.

### *Plasmodium berghei in vivo* and *ex vivo* blood-stage development

For the *in vitro* cultivation of *P. berghei*, blood from highly infected mice (2–5%) was collected and incubated in *Pb* culture medium (RPMI 1640 complemented with 20% heat-inactivated foetal calf serum). The cultures were incubated in a low-oxygen atmosphere (5%) at 37 °C under constant shaking (77 rpm). In order to obtain a synchronized *P. berghei* infection, schizont purification was performed 18 hours after inoculation by a one-step Nycodenz density gradient centrifugation^42^. The obtained schizont pellets were resuspended in medium and intravenously injected into recipient mice for highly synchronized *in vivo* infections. To obtain highly synchronized *ex vivo* cultures, blood from these mice was collected and incubated once more in *Pb* culture medium supplemented with or without different concentrations of Brefeldin A.

Blood-stage propagation of the *exp2-mCherry* (*exp2-mCh*^GFP,res^) and *hsp101-mCherry* (*hsp101-mCh*^GFP,res^) parasite lines was measured by the intravital competition assay as described^20^. This method relies on the co-injection of a double fluorescent mutant line with the YFP-expressing WT (Beryellow) parasite^20^, and their subsequent analysis by flow cytometry.

### Biochemical fractionation and Western blot analysis

Differential solubilisation of *exp2-mCherry* × *GFP*^PV^-infected erythrocytes was performed as described previously^23^. In short, infected erythrocytes were purified on a Nycodenz gradient^47^ and lysed hypotonically for 1 h on ice in 1 mM TRIS-HCl, pH 7.5. Lysates were spun 50 min at 100,000 × *g*. The pellet was resuspended in 1% Triton X-100 in PBS and spun 50 min at 100,000 × *g*.

Equal amounts of each of these fractions or whole protein extracts of mixed blood stages of parasites expressing endogenously tagged proteins, *hsp101-mCherry* (*hsp101-mCh*^GFP,res^) and *exp2-mCherry* (*exp2-mCh*^GFP,res^), were separated on SDS-polyacrylamide and transferred onto a PVDF membrane. Western blotting was performed using a rat monoclonal anti-mCherry antibody (1:5,000; ChromoTek) or a chicken polyclonal anti-GFP antibody (1:5,000; Abcam) and followed by a horseradish peroxidase coupled goat anti-rat/chicken antibody (1:5,000; Jackson ImmunoResearch).

### Light microscopy of live and fixed parasites

Images for live protein localization were recorded on a Zeiss AxioObserver Z1 epifluorescence microscope, equipped with a Zeiss AxioCam MRm camera, and processed minimally with FIJI^48^. Live protein localization was performed only minutes after blood sampling using either conventional slides and coverslips or concanavalin A-coated ibidi μ-Dishes (35 mm, low; Grid500) with pre-warmed *Pb* culture medium. For the assessment of HSP101-mCherry localization during blood-stage development, peripheral blood from a tightly synchronized *hsp101-mCherry* (*hsp101-mCh*^GFP,res^) infection was taken every four hours and diluted 1:50 with *Pb* culture medium. The dilution was then transferred onto a poly-L-lysine coated coverslip. The RBCs were allowed to settle for five minutes at 37 °C prior to fixation with 2.5% glutaraldehyde and 4% paraformaldehyde in PBS for 20 minutes. After repeated washing with PBS, the coverslip was mounted onto a glass slide and protein localization was analysed for 100 parasites per time point. Three-dimensional reconstruction of the EXP2-mCherry signal was performed by optical sectioning with a Zeiss ApoTome.2 using structured illumination technology. Fluorescent membrane labelling was achieved by inoculating an *ex vivo* blood culture with 0.5 mM BODIPY TR ceramide for several hours.

Photobleaching experiments were conducted using *mCherry*^PV^-infected erythrocytes. Red blood cells were suspended in *Pb* culture medium and seeded on a concanavalin A-coated ibidi μ-Dish (35 mm, low; Grid500). After 15 min of incubation at 37 °C, the cells were imaged with a Leica TCS-SP5 confocal microscope at 37 °C using the non-resonant scanner at 1000 Hz. mCherry was excited with the 561 nm laser line. The indicated areas were bleached for 200 ms with the 405 nm laser, before scanning was resumed.

### Electron microscopy

Correlative light and electron microscopy was performed with the *hsp101-mCherry* (*hsp101-mCh*^GFP,res^) parasite line. Infected blood was diluted 1:300 in pre-warmed *Pb* culture medium and the cell suspension was transferred to a concanavalin A coated ibidi μ-Dish (35 mm, low; Grid500) and incubated at 37 °C for 15 minutes. Infected RBCs were continuously imaged with a Zeiss Axiovert 200 M wide field microscope, equipped with a Hamamatsu Orca CCD camera, until subsequent *in situ* fixation with 2.5% glutaraldehyde and washed with PBS. WT ANKA strain parasites were fixed in solution with 2.5% glutaraldehyde in PBS and, after washing with PBS, were embedded in agarose beads. Both WT and *hsp101-mCherry* preparations were contrasted with 0.5% osmium-tetroxide, tannic acid, and 2% uranyl-acetate, dehydrated in a graded ethanol series, cleared in styrene (WT samples only), and infiltrated gradually in several changes of epoxy resin for several hours. The samples were embedded in epoxy on the microscopy dish, using inverted microcentrifuge tubes as moulds, or in standard flat embedding moulds (WT samples), and heat-cured overnight. Sections were made using a diamond knife on a Leica Ultracut-R ultramicrotome. After retrieval on copper grids, the sections were visualized and recorded with a Zeiss LEO 906 or 912 transmission electron microscope, equipped with an SIS-Olympus Morada side mounted or Cantega bottom mount digital camera. For high-resolution modelling, grids of digital images from consecutive sections were stitched and aligned using the TrakEM2 plugin in the FIJI software package^48^,^49^,^50^,^51^. Membranous borders were segmented and aligned interactively and subsequently exported as 3D views.

## Additional Information

**How to cite this article**: Matz, J. M. *et al.* The *Plasmodium berghei* translocon of exported proteins reveals spatiotemporal dynamics of tubular extensions. *Sci. Rep.* **5**, 12532; doi: 10.1038/srep12532 (2015).

## Supplementary Material

Supplementary Information

Supplementary Movie 1

Supplementary Movie 2

Supplementary Movie 3

Supplementary Movie 4

Supplementary Movie 5

Supplementary Movie 6
