# Supplementary material for: The Plasmodium berghei translocon of exported proteins reveals spatiotemporal dynamics of tubular extensions
Source: Sci Rep. 2015 Jul 29;5:12532. doi: 10.1038/srep12532 (PMC4518229; doi:10.1038/srep12532)
Supplement: Supplementary Information [file srep12532-s1.pdf]

Supplementary Information for:

The *Plasmodium berghei* translocon of exported proteins reveals spatiotemporal dynamics of tubular extensions

Joachim M. Matz, Christian Goosmann, Volker Brinkmann, Josephine Grützke, Alyssa Ingmundson, Kai Matuschewski & Taco W.A. Kooij

Content:

- **Supplementary Figure S1 (associated with all main Figures):** Explains the generation of the endogenously tagged transgenic parasite lines for protein localization.
- **Supplementary Figure S2 (associated with Figures 3 and 5):** Explains the generation of transgenic parasite lines with fluorescent marker proteins in the parasitophorous vacuole and endoplasmic reticulum.
- **Supplementary Figure S3 (associated with Figure 1 and Supplementary Figure S1):** HSP101 is trafficked by the parasite's secretory pathway.
- **Supplementary Figure S4 (associated with Figure 3):** FRAP analysis reveals free diffusion from the parasitophorous vacuole to the tubular extensions even after repeated bleaching.
- **Supplementary Figure S5 (associated with Figure 4):** The *P. berghei* membrane-bound tubules demonstrate similarities with the previously described *P. falciparum*-induced tubovesicular network. Co-localization of motile tubules highlighted by GFP^{PV} and a membrane marker highlight this.
- **Supplementary Figure S6 (associated with Figure 4):** Ultrastructure of the vacuolar tubules.
- **Supplementary Figure S7 (associated with Figure 5):** EXP2 localizes to extraparasitic vesicular structures.
- **Supplementary Figure S8 (associated with Figure 6):** Live imaging of four PTEX components in merosomes.
- **Supplementary Video S1 (associated with Figure 1):** Live recording of a *Plasmodium berghei*-induced vacuolar tubule using the *hsp101-mCherry* line. Speed, 4x.
- **Supplementary Video S2 (associated with Figure 3):** FRAP analysis of a vacuolar tubule using the *mCherry*^{PV} line.
- **Supplementary Video S3 (associated with Figure 3):** FRAP analysis with repeated bleaching of a vacuolar tubule using the *mCherry*^{PV} line.
- **Supplementary Video S4 (associated with Figure 4):** 3D-reconstruction of an HSP101-mCherry-positive *Plasmodium berghei*-induced vacuolar tubule.
- **Supplementary Video S5 (associated with Figure 4):** 3D-reconstruction of a wild-type *Plasmodium berghei*-induced vacuolar tubule.
- **Supplementary Video S6 (associated with Figure 5):** 3D-reconstruction of EXP2-mCherry, obtained by optical sectioning of a fixed *exp2-mCherry* trophozoite-infected erythrocyte.
- **Supplementary Table S1 (associated with all main Figures and Supplementary Figures S1 and S2):** Sequences of the primers used to generate the transfection plasmids and evaluate the recombinant parasite lines.
- **Supplementary Table S2 (associated with Figure 4 and Supplementary Figure S4):** To further test the biochemical properties of the *P. berghei* membrane-bound tubules, the effects of different inhibitors on HSP101-mCherry localization were evaluated.

Matz *et al.*, Supplementary Figure S1

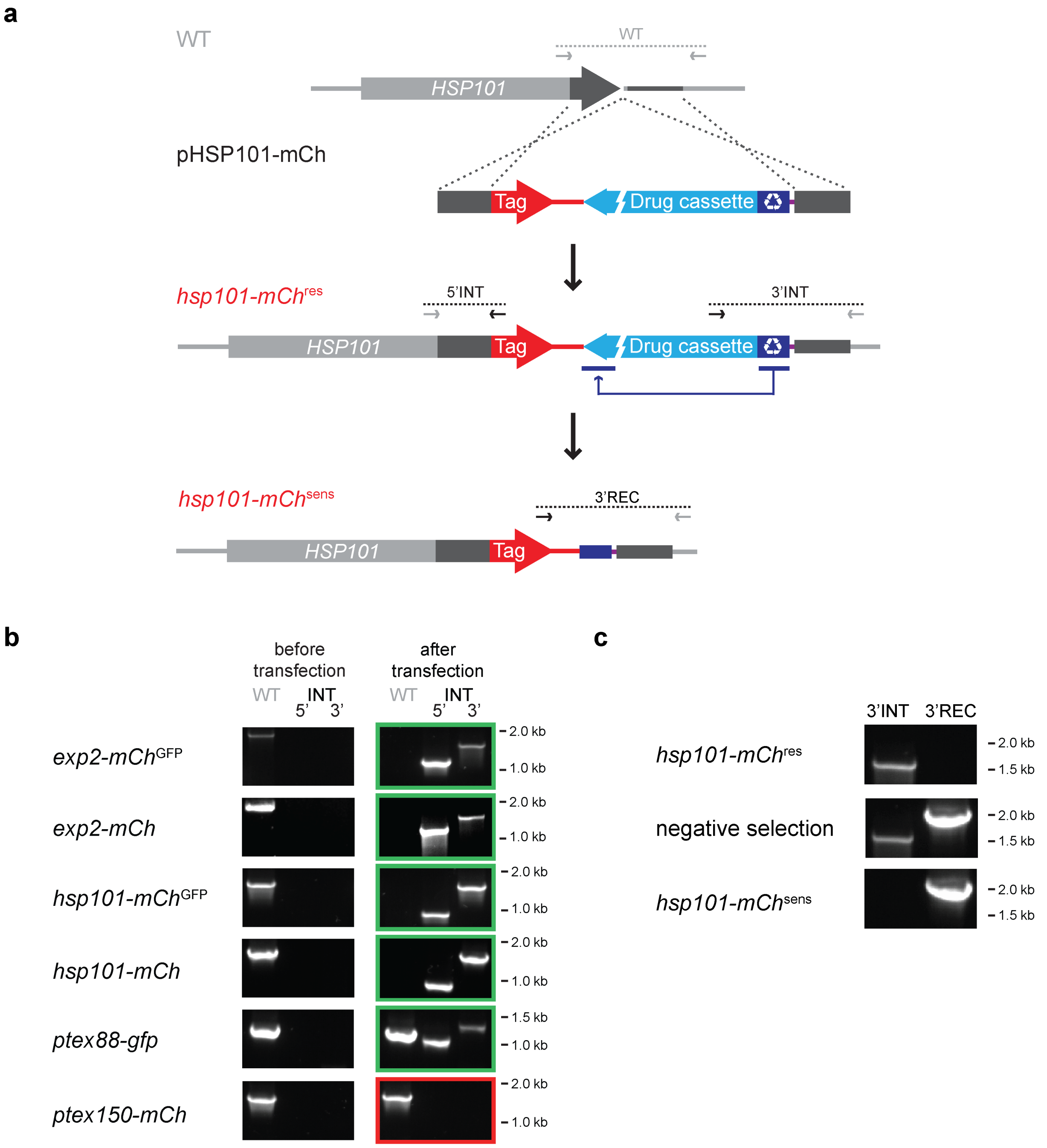

Supplementary Figure S1 | Generation of transgenic parasite lines for protein localization. (a) Recombination strategies for endogenous tagging exemplified by the targeting of *HSP101*. Double crossover integration into the wild-type locus yields transgenic parasites with their endogenous locus tagged by mCherry-3xMyc (red). Recombinant parasites harbour the drug-selectable hDHFR-yFcu cassette (blue) and in some cases a high-expressing GFP-cassette (not shown). Subsequent negative selection with 5-fluorocytosine removes large parts of the drug-selectable cassette. Primer combinations specific for the wild-type locus (WT), integration (5' and 3'INT), and drug cassette recombination (3'REC) are indicated. (b) Diagnostic PCR of the WT loci and integration sites before and after transfection with PTEX component targeting plasmids. Primer combinations were specific for WT, 5' and 3' integration, as indicated in (a). Green and red frames indicate successful and non-successful endogenous tagging, respectively. Note that all generated lines were isolated successfully using flow cytometry with the exception of *ptex88-gfp*, which lacks the highly expressed fluorescent cassette. (c) Genotyping of the *hsp101-mCherry* parasite line before (res, pyrimethamine-resistant) and after negative selection, and after subsequent clonal isolation (sens, pyrimethamine-sensitive). Primer combinations were specific for 3' integration and drug cassette recombination, as indicated in (a).

Matz *et al.*, Supplementary Figure S2

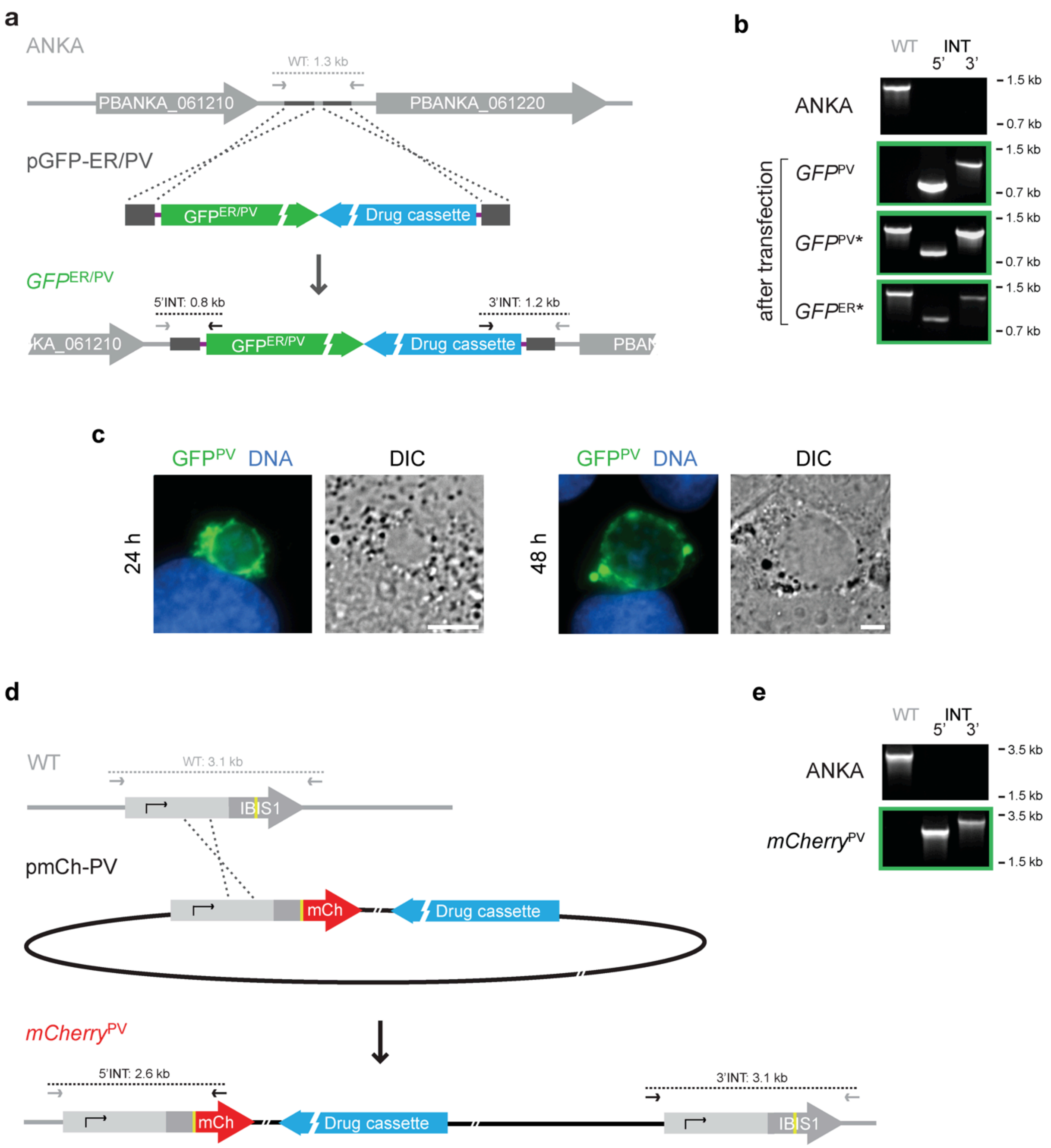

Supplementary Figure S2 | Generation of transgenic parasite lines with fluorescent proteins in the parasitophorous vacuole and endoplasmic reticulum. (a) Recombination strategy for double crossover stable integration of green fluorescent markers of the parasitophorous vacuole (GFP^{PV}; GFP fused to the BiP signal peptide) and the endoplasmic reticulum (GFP^{ER}; GFP fused to the BiP signal peptide and ER retention signal) into the silent intergenic locus on *P. berghei* chromosome 6. In addition to the high-expressing fluorescent protein cassette (green), the recombinant parasites harbour the drug-selectable hDHFR-yFcu cassette (blue). Primer combinations specific for the wild-type locus (WT) and integration (5' and 3'INT) are indicated. (b) Diagnostic PCRs of transgenic parasites. The asterisk marks transfectants for live co-localization, using pyrimethamine-sensitive *hsp101-mCherry* parasites as the recipient strain. (c) Live fluorescent imaging of the GFP^{PV} marker protein reveals a circumferential staining pattern in maturing liver stage parasites, confirming localization to the parasitophorous vacuole. Scale bars, 10 µm. (d-e) Recombination strategy for single crossover integration of a red fluorescent marker of the parasitophorous vacuole ($mCherry^{PV}$) and diagnostic PCRs. Integration yields a fusion of the *IBIS1* N-terminal sequence and the fluorescent mCherry-3xMyc tag. The tag is fused directly adjacent to the PEXEL/VTS motif (yellow) without including a spacer, thereby preventing export.

Matz *et al.*, Supplementary Figure S3

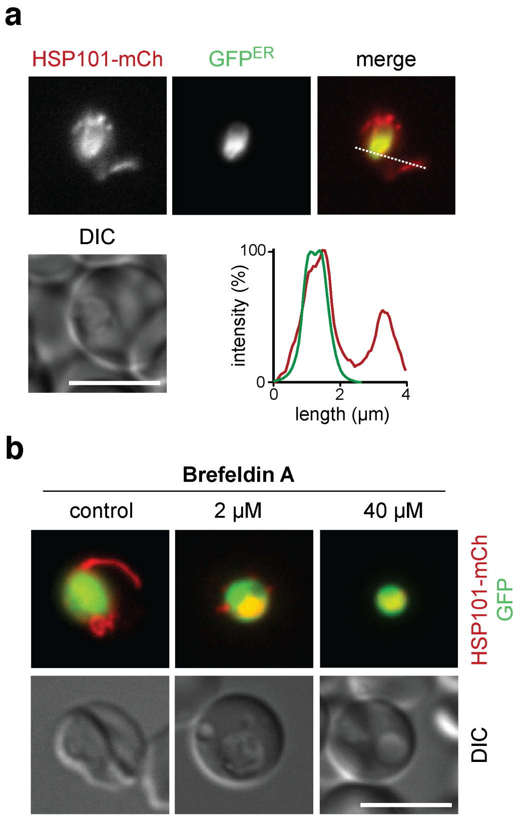

Supplementary Figure S3 | HSP101 is trafficked by the parasite's secretory pathway. (a) Live co-localization of HSP101-mCherry (left) and ER-resident GFP (GFP^{ER}, centre). The line in the merge (right) indicates profiling of the fluorescent signals (bottom left). (b) Localization of HSP101-mCherry and cytoplasmic GFP after treatment with Brefeldin A. Secretion of tagged HSP101 to the tubules is inhibited in a concentration-dependent manner. Synchronized *in vitro* cultures of the *hsp101-mCherry* line were grown in the presence of Brefeldin A and analysed 18 h later. Scale bars, 5 µm.

Matz *et al.*, Supplementary Figure S4

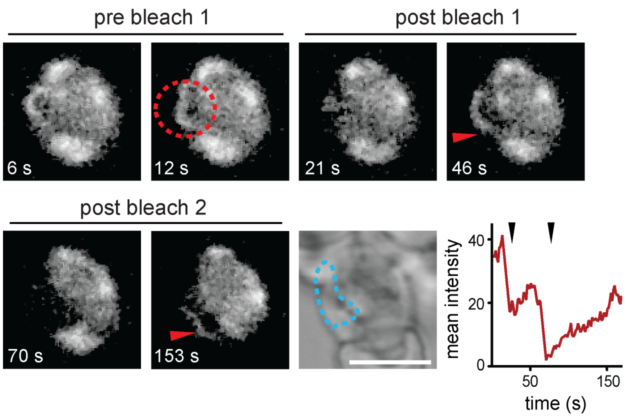

Supplementary Figure S4 | FRAP analysis reveals free diffusion from the parasitophorous vacuole to the tubular extensions. Erythrocytes infected with *mCherry*^{PV} parasites were analysed by confocal microscopy before (pre bleach) and after repeated (post bleach 1 and 2) photo bleaching (red area, bleach location). Shown is a representative trophozoite and the respective temporal fluorescence analysis in the erythrocyte cytoplasm (blue dotted line); black arrowheads indicate times of the bleaching pulses. Scale bar, 5 µm.

Matz *et al.*, Supplementary Figure S5

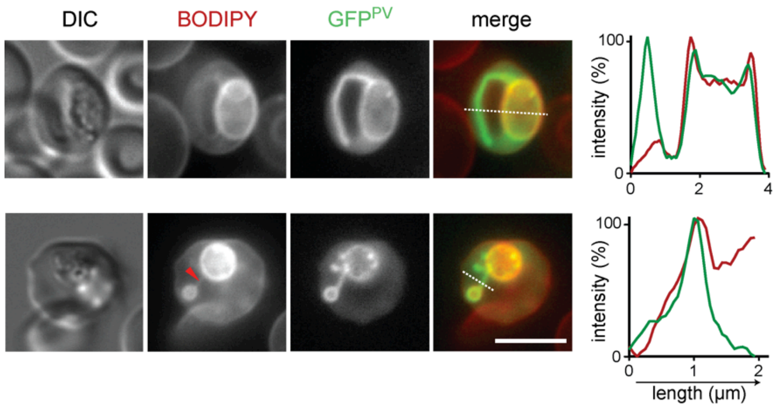

Supplementary Figure S5 | Partial co-localization of motile tubules highlighted by GFP^{PV} and a membrane marker. Co-localization of the lipid marker BODIPY TR ceramide (centre left) and GFP^{PV} (centre). The indicated lines in the merge (centre right) denote profiling of the fluorescent signals (right). Shown are two representative trophozoites. The red arrowhead denotes a vacuolar tubule stained by BODIPY TR ceramide. Scale bar 5 µm.

Matz *et al.*, Supplementary Figure S6

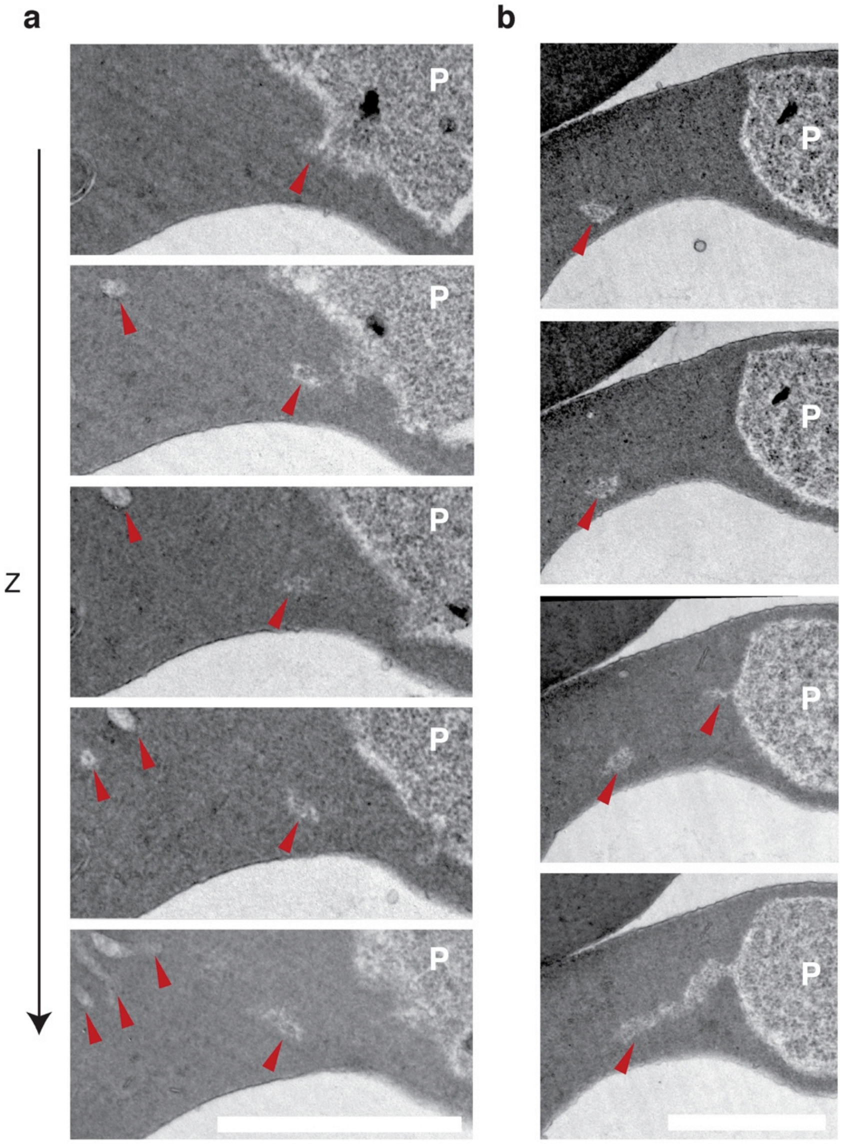

Supplementary Figure S6 | Ultrastructure of the vacuolar tubules. (a,b) Representative high magnification transmission electron micrographs of two WT-infected erythrocytes. The sequential sections show vacuolar tubules (red arrowheads) that emerge from the parasite (P) surface. Scale bars, 1 µm.

Matz *et al.*, Supplementary Figure S7

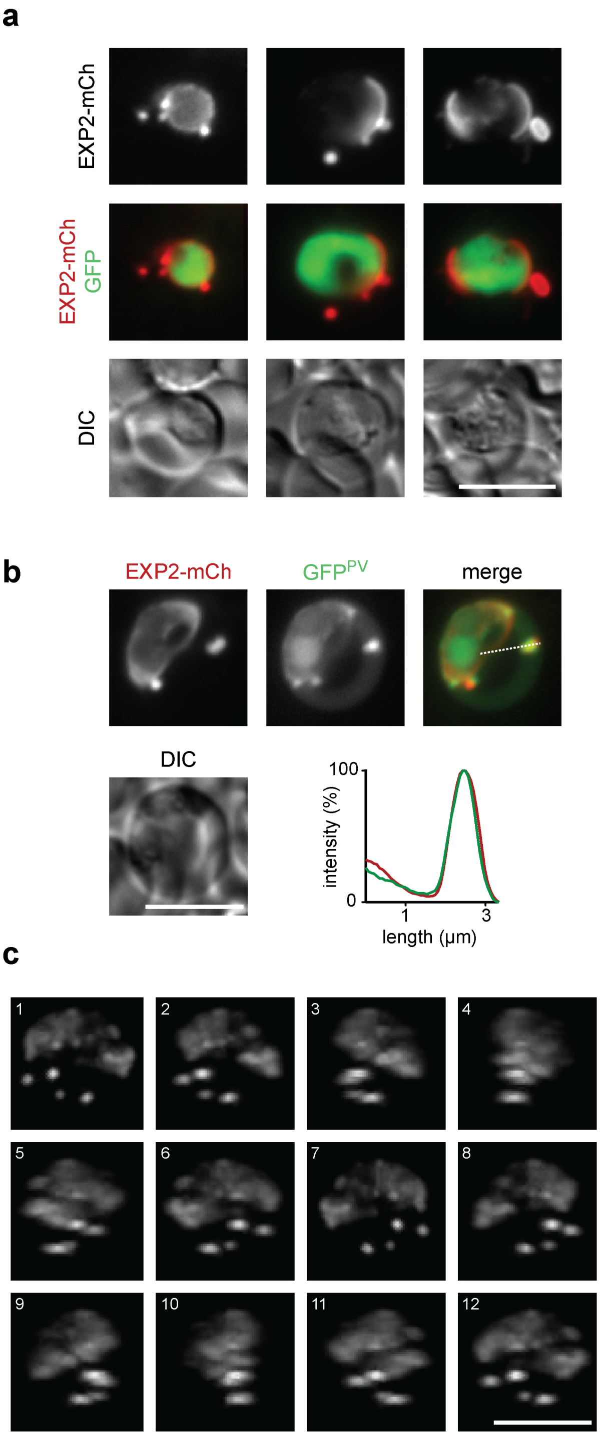

Supplementary Figure S7 | EXP2 localizes to extraparasitic vesicular structures. (a) Fluorescent images of EXP2-mCherry (top) and merge of EXP2-mCherry and cytoplasmic GFP (middle). Three representative trophozoites are shown. (b) Live co-localization of EXP2-mCherry (top left) with a marker protein of the parasitophorous vacuole (GFP^{PV}; top centre). The indicated line in the merge (top right) denotes profiling of the fluorescent signals (bottom right). (c) 12 frames of Supplementary Video S6 showing a 3D-reconstruction of EXP2-mCherry, obtained by optical sectioning of a fixed *exp2-mCherry* parasite-infected erythrocyte. Scale bars, 5 µm.

Matz *et al.*, Supplementary Figure S8

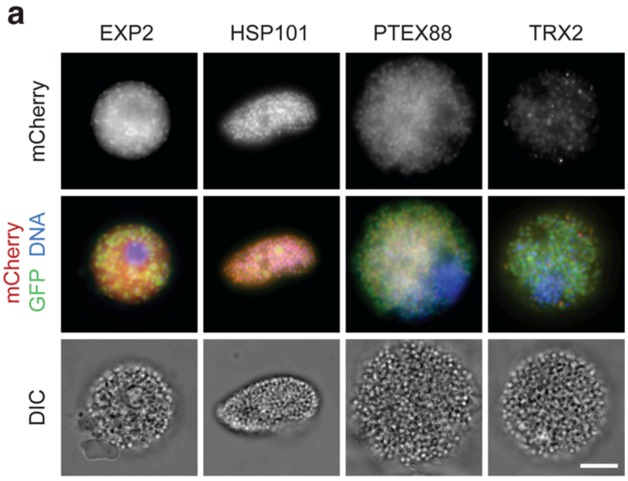

Supplementary Figure S8 | Live imaging of four PTEX components in merosomes. Micrographs of the merozoite containing merosomes derived from *in vitro* cultured liver stage parasites 72 h after infection. Shown are representative images including the fluorescent signal of the tagged protein (top), a merge of tagged protein, cytoplasmic GFP, and Hoechst 33342 DNA dye (middle) and differential interference contrast images (DIC, bottom). Scale bar, 10 µm.

Matz *et al.,* Supplementary Table S1 | Primer sequences.

Primer Name	Primer Sequence (restriction sites underlined)	Size WT (bp)[a]	Size INT (bp)[b]	Use[c]	Target	Reference
SIL6F	GACAGCGCATATGATGGATG	1315	847	GT	PbSIL6	(Kenthirapalan, 2012)
SIL6R	TACGAATACGCAATTTCTCAAAC		1247	GT	PbSIL6	(Kenthirapalan, 2012)
mCherryFor	CTATACCATCGTGGAACAGTAC			GT	mCherry	
mCherryRev1	CCCTCCATGTGAACCTTGAAG			GT	mCherry	(Haussig, 2011)
mCherryRev2	GATCCTTACTTGTACAGC			GT	mCherry	
GFPrev	TGTGCCCATTAACATCACCATC			GT	GFP	(Haussig, 2013)
5’HSP70rev	CAATTTGTTGTACATAAAATAGGCAG			GT	5'PbHSP70	(Kenthirapalan, 2012)
5’DHFRrev	ATGAAATACCGCTCCATTTTTCC			GT	5'PbDHFR-TS	(Kenthirapalan, 2012)
BiP-SP-GFP-F-SwaI	GGGATTTAAATATGGGAAATTCAAAGGCATTTGTTTTAGTATTATTTGT ATCCCTGTTGAAATTTATAAGCGCCGGACATATGTCTGTGAGTAAAGG	830		TV	GFP	
BiP-RS-GFP-R-BamHI	ATTGGATCCTTATAATTCATCACTGGCGCCTTTGTATAGTTCATC			TV	GFP	
GFP-R-BamHI-KasI	CTAGGATCCTTAGGCGCCTTTGTATAGTTCATCCATGCCATGTGTAATC CCTGCTGCTG	818		TV	GFP	
CT-EXP2-F-SacII	AATAATCCGCGGTTAAGGTGGTCTCGTATGTGGTGG	703		TV	CT-PbEXP2	
CT-EXP2-R-HpaI	AATAATGTTAACAGCCTCATTAGAATCAGTTTCTTGC			TV	CT-PbEXP2	
3'EXP2-F-XhoI	AGTCCACTCGAGCTAAATAGAGAAACAATGGTGTTTTATAAGC	606		TV	3'PbEXP2	(Matz, 2013)
3'EXP2-R-KpnI	AGGGCTGGTACCTTTATTGAAAATGCAAAATAACGAAAATAGC			TV	3'PbEXP2	(Matz, 2013)
CT-EXP2-F	GATTTAGCAGCAACCACTGCC	1998	1049	GT	CT-PbEXP2	
3'EXP2-R	TTGGCATGTGGCAATAAGCATAC		1475	GT	3'PbEXP2	(Matz, 2013)
CT-HSP101-F-SacII	ATTATTCCGCGGGACCTCATTCTGTTGTTCTATTTGATG	638		TV	CT-PbHSP101	
CT-HSP101-R-NaeI	ACACTTGCCGGCTGACAATGAAAGGTTAATAACAATGTTGTTG			TV	CT-PbHSP101	
3'HSP101-F-XhoI	AGATGTCTCGAGTTAAATAAAACAAACACGATATGTTGCATG	848		TV	3'PbHSP101	(Matz, 2013)
3'HSP101-R-KpnI	TTACTTGGTACCTTATTATCACACACTTTTTCATAGATATTGC			TV	3'PbHSP101	(Matz, 2013)
CT-HSP101-F	GTCAGAATTTACAGAAGCACATTCAG	1674	828	GT	CT-PbHSP101	
3'HSP101-R	CGTGTGGGCATAGATCAGTGA		1547 (res)<br>2058 (sens)	GT	3'PbHSP101	(Matz, 2013)
CT-PTEX88-F	GCTTGATGAAATATGCTATTATGATTCTC	1196	1025	GT	CT-PbPTEX88	(Matz, 2013)
3'PTEX88-R	GTGACTTGGATTCAGATTAAAAATGCA		1285	GT	3'PbPTEX88	(Matz, 2013)
CT-PTEX150-F-SacII	ACCATACCGCGGCTATTATCATCAAGCACCACAGTTG	622		TV	CT-PbPTEX150	
CT-PTEX150-R-HpaI	TTATTGTTAACTTCATCTTCATCTTCATCCTCTGG			TV	CT-PbPTEX150	
3’PTEX150-F-XhoI	AACGTTCTCGAGTAGCATAGGTGCGCGAGTC	790		TV	3’PbPTEX150	(Matz, 2013)
3’PTEX150-R-KpnI	TTAGTGGGTACCGGTAAGAACAAGAACAAAAATTGCATTATC			TV	3’PbPTEX150	(Matz, 2013)
CT-PTEX150-F	GATGAAAACTTTTACGATGCTTACAAAC	1645	770	GT	CT-PbPTEX150	
3’PTEX150-R	TGCAAGCATTGTGTACCATATTAACC		1486	GT	3’PbPTEX150	(Matz, 2013)
5'IBIS1-F-SacII	GCATCCGCGGCGTATTTTAATCATACACTATACGTTTTTCC	1784		TV	5'PbIBIS1	
IBIS1-PEXEL-R-SpeI	GGACTAGTATCCAACTCTGATAATATTCTGCTTTTTCC			TV	PEXEL-PbIBIS1	
5'IBIS1-F	GATCCTTACTTGTACAGC	3147	2579	GT	5'PbIBIS1	
3'IBIS1-R	TCCAACTCTGATAATATTCTGCTTTTTCC		3086	GT	3'PbIBIS1	

[a] Sizes of the PCR products of forward and reverse primers on WT gDNA.

[b] Sizes of the respective integration-specific PCR products; forward 5’ gene-specific primers combined with 5’HSP70rev, mCherryRev1/2, or GFPrev and reverse 3’ gene-specific primers combined with 5’DHFRrev (pyrimethamine-resistant lines), mCherryFor (pyrimethamine-sensitive lines), or T7 (*mCherry*[PV]).

[c] TV, primers used for construction of Transfection Vectors; GT, primers used for GenoTyping.

REFERENCES

Haussig, J.M., Matuschewski, K., Kooij, T.W.A. (2011). Inactivation of a *Plasmodium* apicoplast protein attenuates formation of liver merozoites. Mol. Microbiol. *81*, 1511-1525.

Haussig, J.M., Matuschewski, K., Kooij, T.W.A. (2013). Experimental genetics of *Plasmodium berghei* NFU in the apicoplast iron-sulfur cluster biogenesis pathway. PLoS ONE *8*, e67269.

Kenthirapalan, S., Waters, A. P., Matuschewski, K., and Kooij, T. W. A. (2012). Flow cytometry-assisted rapid isolation of recombinant *Plasmodium berghei* parasites exemplified by functional analysis of aquaglyceroporin. Int. J. Parasitol. *42*, 1185–1192.

Matz, J. M., Matuschewski, K., and Kooij, T. W. A. (2013). Two putative protein export regulators promote *Plasmodium* blood stage development *in vivo*. Mol. Biochem. Parasitol. *191*, 44–52.

Matz *et al.*, Supplementary Table S2 | The effects of different inhibitors on HSP101-mCherry localization.

Inhibitor	Target	Concentrations	HSP101-mCh in tubule	Tubular motility	Developmental delay
cytochalasin D	actin polymerization (▼)	200 nM	+	+	–
		20 µM	+	+	–
		50 µM	+	+	+/–
jasplakinolide	actin polymerization (▲)	1 nM	+	+	–
		10 nM	+	+	–
		500 nM	+	+	+/–
blebbistatin	myosin	2 nM	+	+	–
		200 nM	+	+	–
		20 µM	+	+	–
nocodazole	tubulin polymerization	300 nM	+	+	–
		30 µM	+	+	+/–
		300 µM	+	+	+
EHNA	dynein	20 nM	+	+	–
		200 nM	+	+	+/–
		2 µM	+	+	+
vanadate	ATPase domains	20 nM	+	+	+/–
		200 nM	+	+	+
		2 µM	+	+	+
PPMP	sphingomyelin synthase	500 nM	+	+	+/–
		5 µM	+	+	+
		50 µM	+	+	+

EHNA, erythro-9-(2-hydroxy-3-nonyl)adenine; PPMP, DL-threo-1-Phenyl-2-palmitoylamino-3-morpholino-1-propanol. Experiments have been performed with synchronized and unsynchronized *in vitro* cultures of the *hsp101-mCherry* parasite line.
